# Supplementary material for: Genomic epidemiology of Vibrio cholerae during a mass vaccination campaign of displaced communities in Bangladesh
Source: Nat Commun. 2023 Jun 24;14:3773. doi: 10.1038/s41467-023-39415-3 (PMC10290697; doi:10.1038/s41467-023-39415-3)
Supplement: Supplementary file 1 — Supplementary Information [file 41467_2023_39415_MOESM1_ESM.pdf]

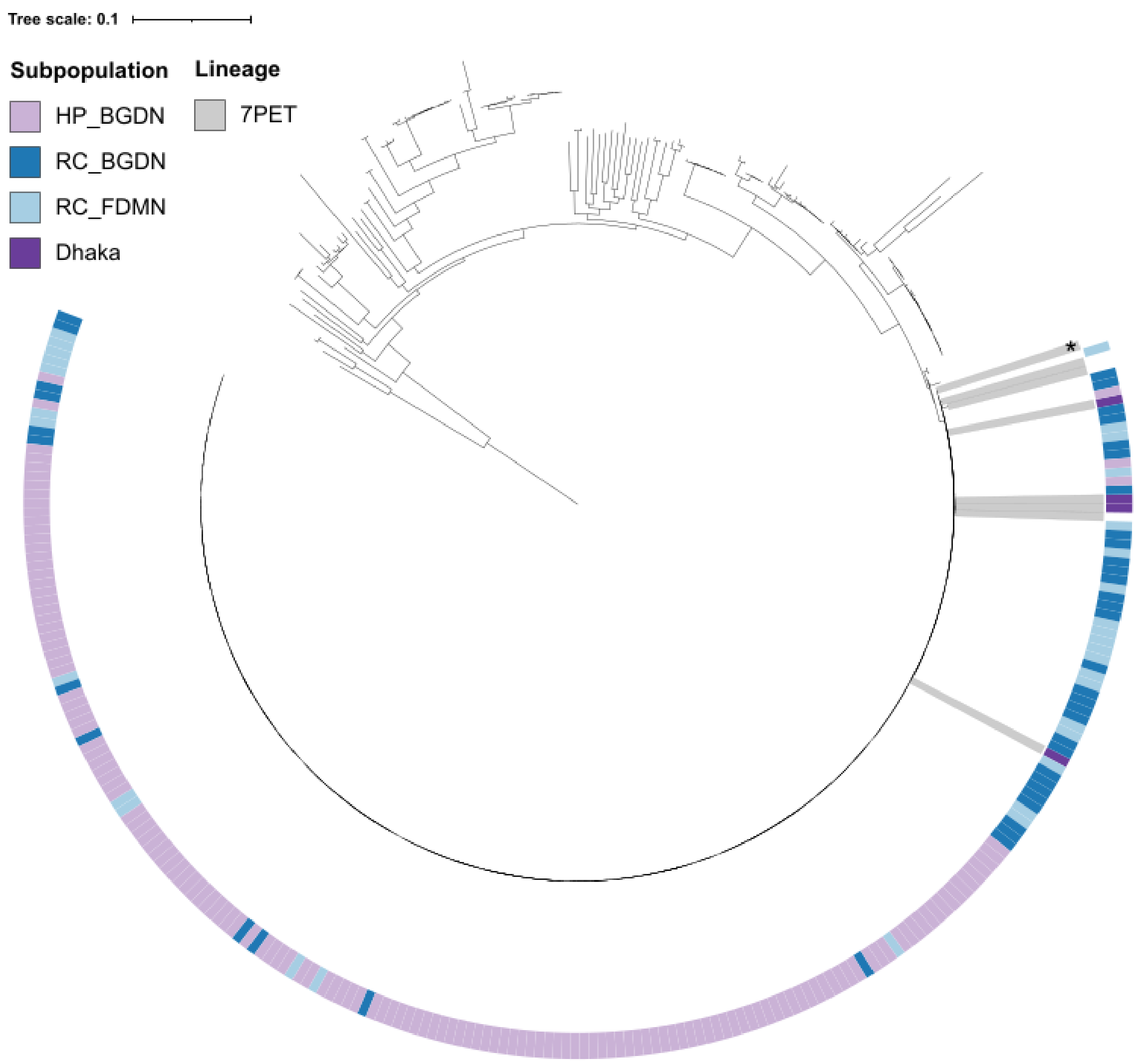

Supplementary Figure S1: ***V. cholerae* core genome single nucleotide variant (SNP) phylogeny.** A maximum likelihood phylogeny was constructed using SNPs (*n* = 490,634) from the alignment of genes core to the 223 genomes sequenced in this study and 148 *V. cholerae* genomes representative of the diversity within the species (*n* = 2,834 genes). Grey shading on the tree tips depicts the 7PET lineage, with the commonly used reference genome N16961 denoted by an asterisk, whilst the coloured strip denotes subpopulations from which our samples were isolated (see key). The tree is midpoint-rooted and the scale bar represents substitutions per site.

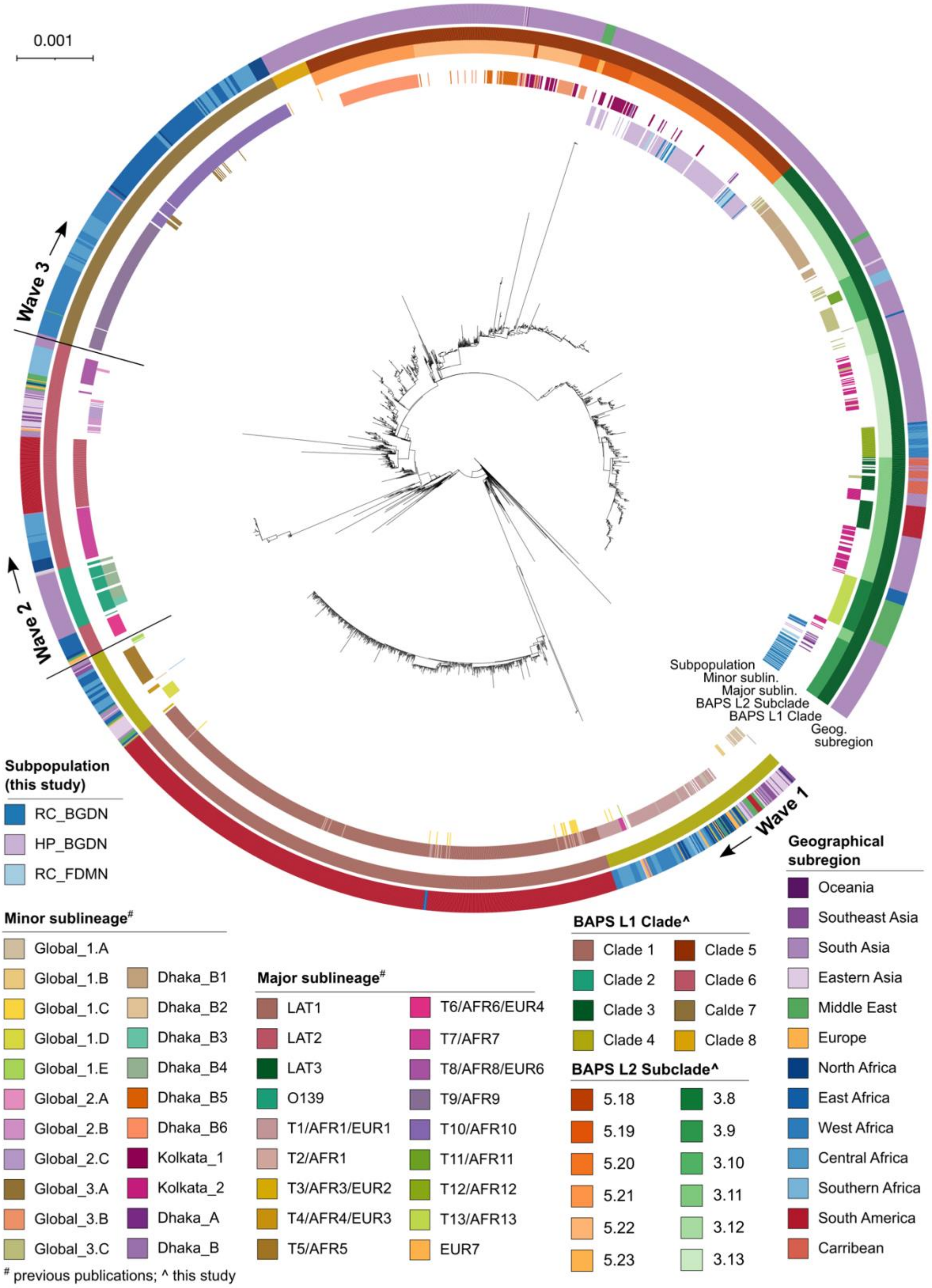

Supplementary Figure S2: ***V. cholerae* 7PET whole genome single nucleotide variant (SNP) phylogeny.** Whole genome SNP maximum-likelihood outgroup-rooted phylogenetic tree of 223 *V. cholerae* strains from Cox's Bazar from this study and

2,383 published genomes. Coloured strips denote geographical subregion, major and minor sublineages from previous studies, hierBAPS clade and subclade, and the subpopulations from which our samples were isolated (see key).

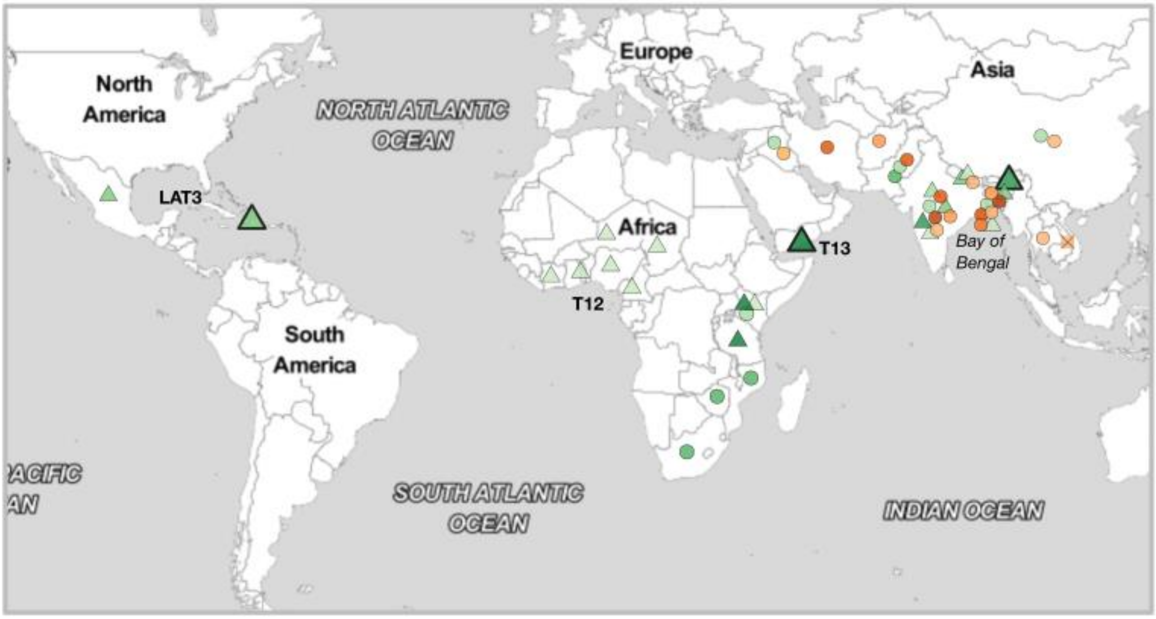

Supplementary Figure S3: Contrast of the geographical distribution of subclades. Genomes from Clade 3 (green shades; global distribution) and Clade 5 (orange shades; Asian distribution) are shown. Sites of cholera outbreaks associated with humanitarian crises are marked with thick outlines. Distribution of selected previously documented major lineages are also labelled. See key in Supplementary Figure S2. Maps were obtained via StamenMaps from Stamen Design (www.maps.stamen.com/toner), under a Creative Commons Attribution (CC BY 3.0) license (https://creativecommons.org/licenses/by/3.0/) and plotted using the R package ggmap.

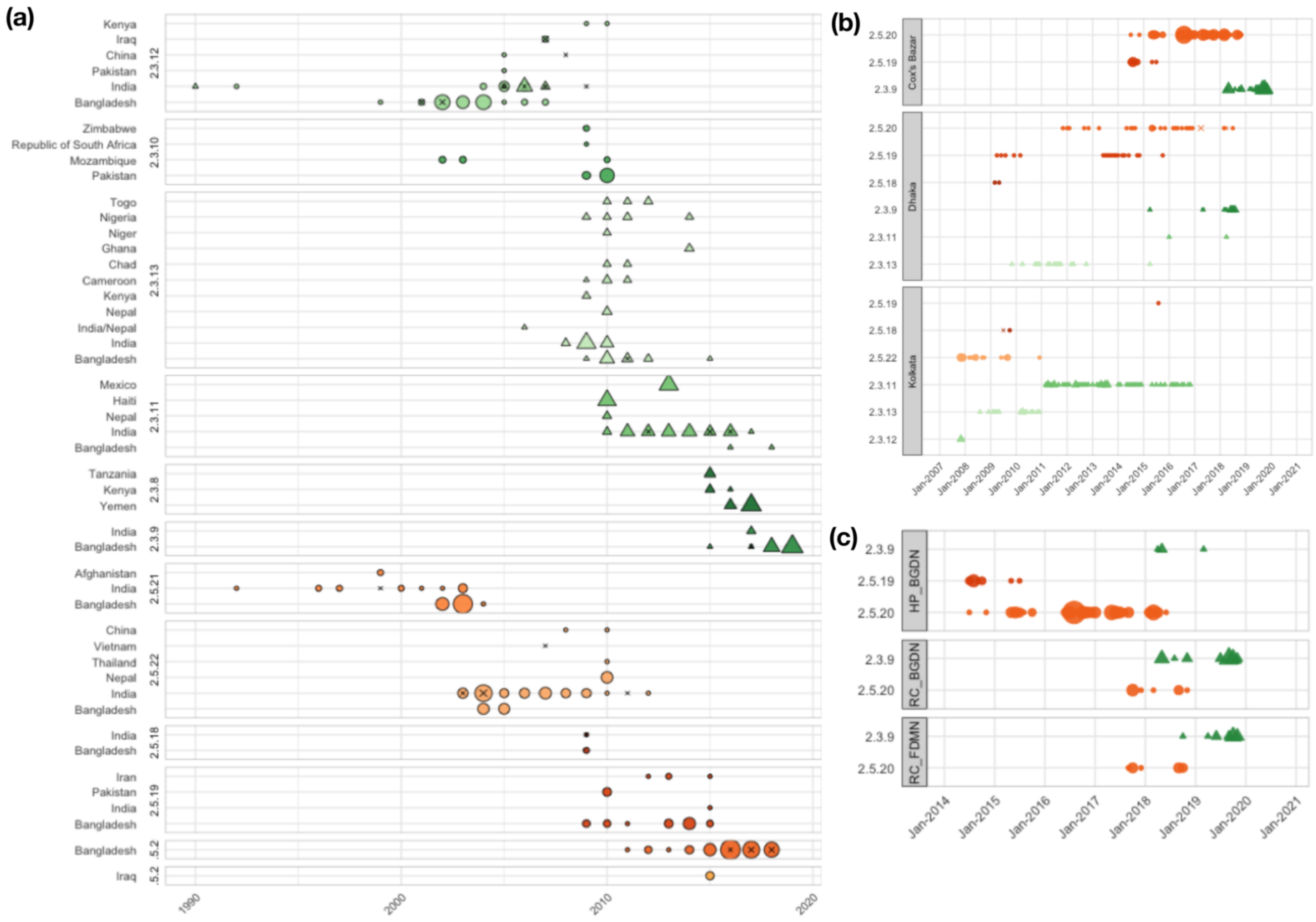
(a)
Kenya
Iraq
China
Pakistan
India
Bangladesh
2.3.12
Zimbabwe
Republic of South Africa
Mozambique
Pakistan
2.3.10
Togo
Nigeria
Niger
Ghana
Chad
Cameroon
Kenya
Nepal
India/Nepal
India
Bangladesh
2.3.13
Mexico
Haiti
Nepal
India
Bangladesh
2.3.11
Tanzania
Kenya
Yemen
2.3.8
India
Bangladesh
2.3.9
Afghanistan
India
Bangladesh
2.5.21
China
Vietnam
Thailand
Nepal
India
Bangladesh
2.5.22
India
Bangladesh
2.5.18
Iran
Pakistan
India
Bangladesh
2.5.19
Bangladesh
.5.2
Iraq
.5.2
1990
2000
2010
2020
(b)
Cox's Bazar
2.5.20
2.5.19
2.3.9
Dhaka
2.5.20
2.5.19
2.5.18
2.3.9
2.3.11
2.3.13
Kolkata
2.5.19
2.5.18
2.5.22
2.3.11
2.3.13
2.3.12
Jan-2007
Jan-2008
Jan-2009
Jan-2010
Jan-2011
Jan-2012
Jan-2013
Jan-2014
Jan-2015
Jan-2016
Jan-2017
Jan-2018
Jan-2019
Jan-2020
Jan-2021
(c)
HP_BGDN
2.3.9
2.5.19
2.5.20
RC_BGDN
2.3.9
2.5.20
RC_FDMN
2.3.9
2.5.20
Jan-2014
Jan-2015
Jan-2016
Jan-2017
Jan-2018
Jan-2019
Jan-2020
Jan-2021

Supplementary Figure S4: **Temporal and geographic distribution of subclades.** Plots depict the distribution of subclades globally **(a)**, in the Bay of Bengal **(b)** and Cox’s Bazar **(c)**. Genome counts are denoted by the size of the point, while the shape denotes the *ctxB* type (see key).

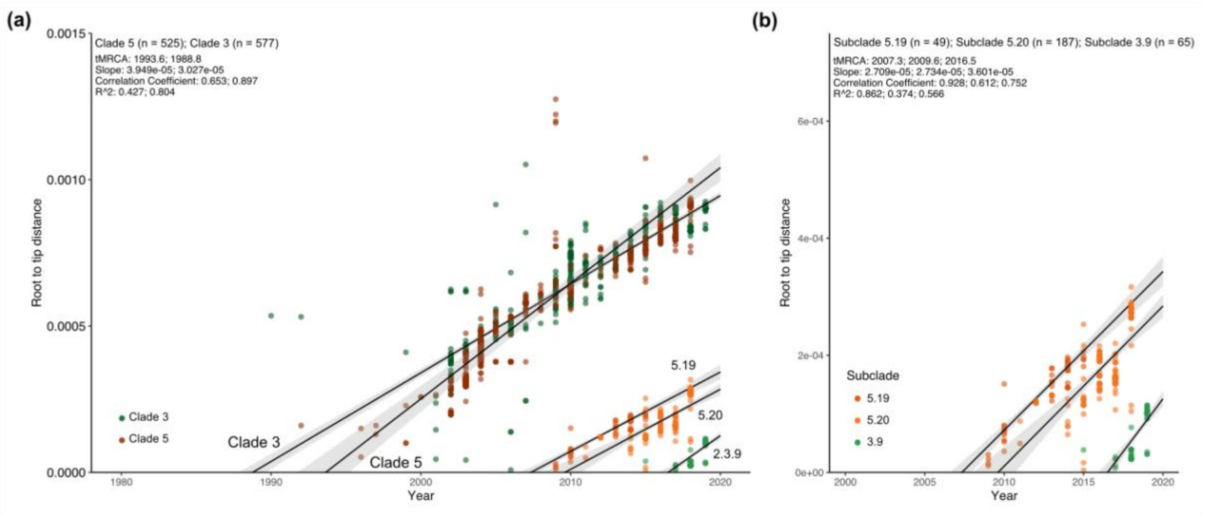

Supplementary Figure S5: **Evolutionary dynamics of Clades 3 and 5.** Plots depict the relationship between root-to-tip distance on the phylogeny and year of isolation (linear regression representing the mean +/- standard error depicted by grey shading) for clades 3 and 5 **(a)** and their subclades present in Cox's Bazar **(b)**. Points are coloured by clade or subclade (see key). Note the different axis ranges for (a) and (b).

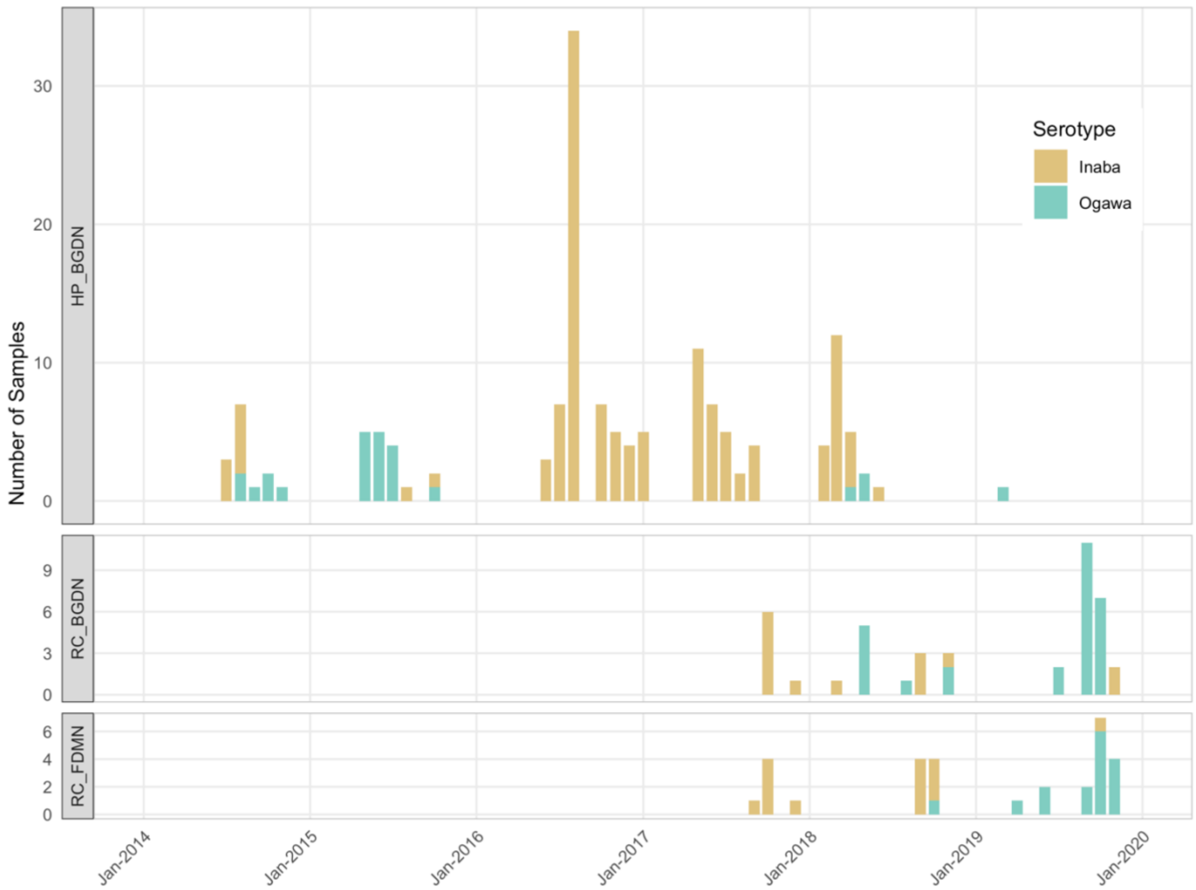

Supplementary Figure S6: Serotype switching over time in the Cox's Bazar subpopulations. Number of samples per month are plotted over time. Bars are coloured by serotype (see key). Note the different y axis ranges for the different subpopulations.

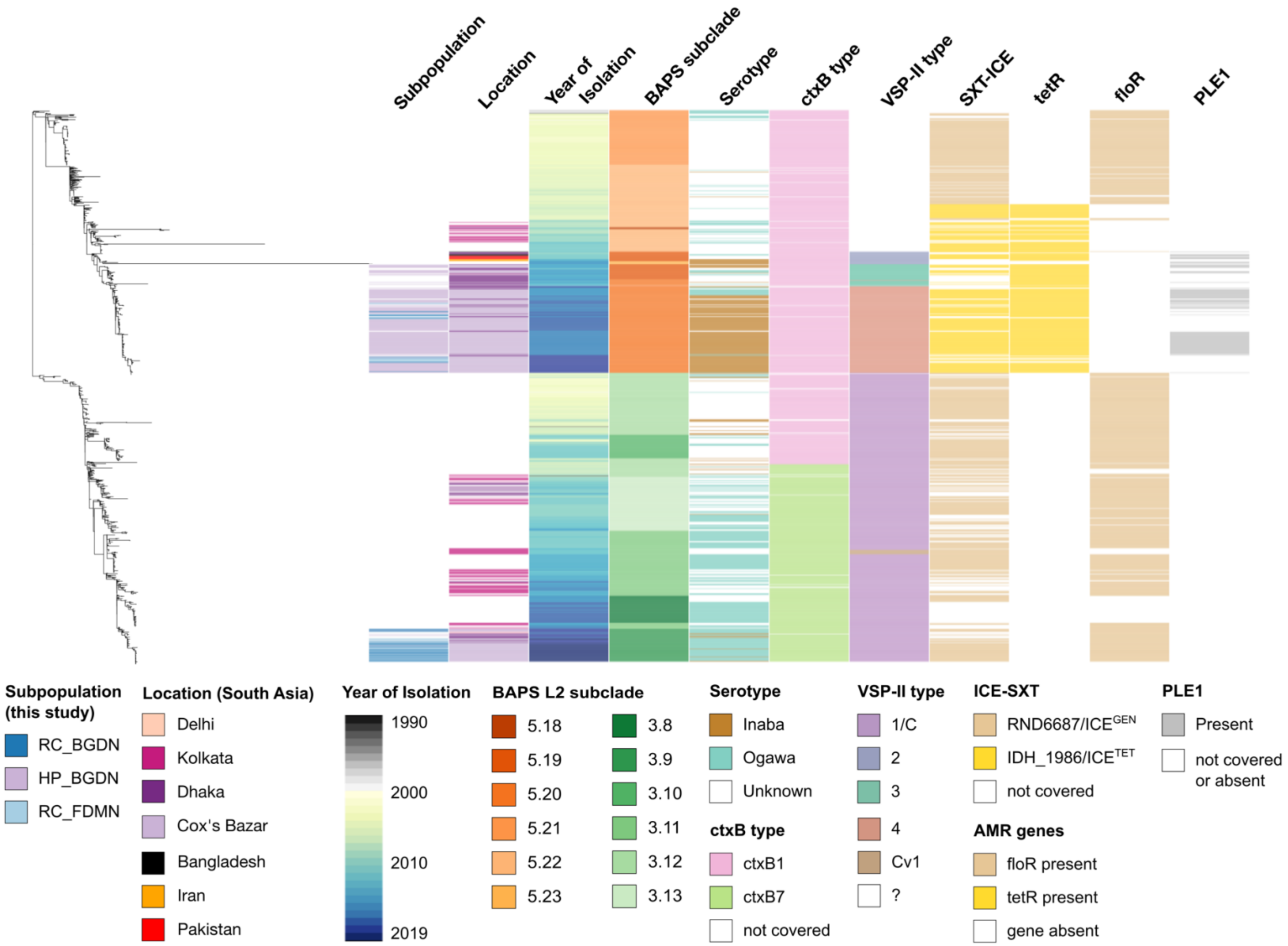

Subpopulation
Location
Year of Isolation
BAPS subclade
Serotype
ctxB type
VSP-II type
SXT-ICE
tetR
floR
PLE1
Subpopulation (this study)
RC_BGDN
HP_BGDN
RC_FDMN
Location (South Asia)
Delhi
Kolkata
Dhaka
Cox's Bazar
Bangladesh
Iran
Pakistan
Year of Isolation
1990
2000
2010
2019
BAPS L2 subclade
5.18
5.19
5.20
5.21
5.22
5.23
3.8
3.9
3.10
3.11
3.12
3.13
Serotype
Inaba
Ogawa
Unknown
ctxB type
ctxB1
ctxB7
not covered
VSP-II type
1/C
2
3
4
Cv1
?
ICE-SXT
RND6687/ICE^GEN
IDH_1986/ICE^TET
not covered
AMR genes
floR present
tetR present
gene absent
PLE1
Present
not covered or absent

Supplementary Figure S7: Genetic microvariation within *V. cholerae* clades present in Cox's Bazar. A subtree of the *V. cholerae* 7PET phylogeny containing PG 1.5 and PG 1.3 plotted against selected metadata and gene presence. Coloured strips denote the subpopulation, location, year of isolation, subclade, serotype, ctxB type, VSP-II type, SXT-ICE type, *tet*R and *flo*R genes, and PLE1 presence (see key).
