# Supplementary material for: Genomic epidemiology of Vibrio cholerae during a mass vaccination campaign of displaced communities in Bangladesh
Source: Nat Commun. 2023 Jun 24;14:3773. doi: 10.1038/s41467-023-39415-3 (PMC10290697; doi:10.1038/s41467-023-39415-3)
Supplement: Supplementary file 3 — Description of Additional Supplementary Files [file 41467_2023_39415_MOESM3_ESM.pdf]

Description of Additional Supplementary Files

File Name: Supplementary Data 1
Description: Metadata and accession information for genomes included in 7PET phylogenetic tree, including both previously published genomes and those sequenced in this study.

File Name: Supplementary Data 2
Description: Metadata and accession information for contextual published genomes included in *V. cholerae* species phylogenetic tree.

File Name: Supplementary Data 3
Description: Accession information for published gene and region sequences used for custom BLAST databases.
