# Supplementary material for: Genomic epidemiology of Vibrio cholerae during a mass vaccination campaign of displaced communities in Bangladesh
Source: Nat Commun. 2023 Jun 24;14:3773. doi: 10.1038/s41467-023-39415-3 (PMC10290697; doi:10.1038/s41467-023-39415-3)
Supplement: Supplementary file 7 — Reporting Summary [file 41467_2023_39415_MOESM7_ESM.pdf]

nature portfolio

Corresponding author(s): Alyce Taylor-Brown, Nicholas Thomson, Firdausi Qadri

Last updated by author(s): 22/05/2023

Reporting Summary

Nature Portfolio wishes to improve the reproducibility of the work that we publish. This form provides structure for consistency and transparency in reporting. For further information on Nature Portfolio policies, see our Editorial Policies and the Editorial Policy Checklist.

Statistics

For all statistical analyses, confirm that the following items are present in the figure legend, table legend, main text, or Methods section.

n/a	Confirmed	
☐	☒	The exact sample size (*n*) for each experimental group/condition, given as a discrete number and unit of measurement
☒	☐	A statement on whether measurements were taken from distinct samples or whether the same sample was measured repeatedly
☐	☒	The statistical test(s) used AND whether they are one- or two-sided<br>*Only common tests should be described solely by name; describe more complex techniques in the Methods section.*
☐	☒	A description of all covariates tested
☒	☐	A description of any assumptions or corrections, such as tests of normality and adjustment for multiple comparisons
☐	☒	A full description of the statistical parameters including central tendency (e.g. means) or other basic estimates (e.g. regression coefficient) AND variation (e.g. standard deviation) or associated estimates of uncertainty (e.g. confidence intervals)
☐	☒	For null hypothesis testing, the test statistic (e.g. *F*, *t*, *r*) with confidence intervals, effect sizes, degrees of freedom and *P* value noted<br>*Give P values as exact values whenever suitable.*
☒	☐	For Bayesian analysis, information on the choice of priors and Markov chain Monte Carlo settings
☒	☐	For hierarchical and complex designs, identification of the appropriate level for tests and full reporting of outcomes
☒	☐	Estimates of effect sizes (e.g. Cohen's *d*, Pearson's *r*), indicating how they were calculated

Our web collection on statistics for biologists contains articles on many of the points above.

Software and code

Policy information about availability of computer code

Data collection	No software was used for data collection.
Data analysis	We assembled and annotated the genome assemblies using Velvet v1.2.10, Prokka v1.5, respectively. The pangenome was determined using Roary v3.13.0. Phylogenetic trees were constructed with IQ-TREE v1.6.10, based on polymorphic sites in the core genome alignment obtained using snp-sites v2.5.1. For a reference-mapped alignment, variants were called against a reference sequence using SMALT v0.7.4, Picard v1.92 and samtools mpileup v0.1.19. Phylogenetic trees were visualised with Phandango v1.3.0 and iTOL v5 and v6. fastBAPS v1.0.3 was run on the tree to determined clade-level clusters, and TempEst v1.5.3 was run to determine a temporal signal in the tree. We assigned AMR genes and ctxB types using ARIBA v2.14.4 and BLAST, the latter of which was also used to determine the present of the SXT-ICE and PLE. R packages used were ape, dplyr, ggplot2 and ggmap,and statistical analysis was conducted in base R.

For manuscripts utilizing custom algorithms or software that are central to the research but not yet described in published literature, software must be made available to editors and reviewers. We strongly encourage code deposition in a community repository (e.g. GitHub). See the Nature Portfolio guidelines for submitting code & software for further information.

Data

Policy information about availability of data

All manuscripts must include a data availability statement. This statement should provide the following information, where applicable:

- Accession codes, unique identifiers, or web links for publicly available datasets
- A description of any restrictions on data availability
- For clinical datasets or third party data, please ensure that the statement adheres to our policy

The read data generated in this study have been deposited in the ENA database under accession codes ERS3328687 to ERS4218290 (for example, https://www.ebi.ac.uk/ena/browser/view/ERS3328750), as provided in Supplementary data file S1. The metadata for this study is also provided in Supplementary data file S1. The sequence accession codes and metadata for previously published genomes is provided in Supplementary data file S1 and S2. The Comprehensive Antibiotic Resistance Database (CARD) database is available at https://card.mcmaster.ca/.

Human research participants

Policy information about studies involving human research participants and Sex and Gender in Research.

Reporting on sex and gender	Data on sex was collected but sparsely reported: "The number of female and male patients were 118 and 105, respectively. Eighteen samples included here were from patients who developed symptoms after being vaccinated during the MVC. Of these, 12 were females between two and 57 years old and six were males under five years old."
Population characteristics	Our samples were obtained from two populations, which we subdivided into three subpopulations: "We sequenced isolates obtained from 150 Bangladeshi Nationals (BGDNs) residing outside of, but adjacent to the refugee camps (RCs) (host population; HP_BGDN) and from 42 BGDNs and 31 Forcibly Displaced Myanmar Nationals (FDMNs) residing in the RCs (RC_BGDN and RC_FDMN, respectively; Supp Tables S1 and S2).<br><br>Our samples were obtained over a four year surveillance period: "Both sample sets spanned pre-, during, and post-MVC periods, with host population BGDN samples collected from July 2014 until March 2019, and the RC samples collected over a shorter but more recent time frame, September 2017 to November 2019"...<br><br>The age range of the cohort from which our samples came was 3 months old to 75 years old: "The majority of the patients included in this study were adults over 18 years old (n = 135); 25 patients were aged from 5 to 18, and 62 were under 5 years old (Supp Table S1)."<br><br>The included population all presented with acute watery diarrhoea. Some were treated with antibiotics and some with ORS (at the discretion of the physician). Some of out samples were from patients that recieved the oral choelra vaccine during the Mass Vaccination Campaign: "Eighteen samples included here were from patients who developed symptoms after being vaccinated during the MVC. Of these, 12 were females between two and 57 years old and six were males under five years old. All were from FDMNs residing in RCs in Balukhali, Kutupalang, Nayapara and Teknaf, except for one who was a BGDN residing in the Kutupalang RC. The vaccination status of 10 patients included in this study (six RC_BGDNs and four FDMNs) was unknown, and no BGDNs residing in the adjacent host communities were known to be vaccinated prior to symptom onset"
Recruitment	"Samples utilised in this study were obtained through nationwide diarrhoeal disease sentinel surveillance described in Khan et al. (2020) and Qadri et al. (2018)."<br>"Patients were recruited if they met the AWD case definition and hence were suspected cholera case."... "Four patients who met the case definition and and had no other severe comorbidity were enrolled by the physician from Saturday to Wednesday each week. Two patients with diarrhoea aged less than five years old and two patients aged five years or older were enrolled each day; if the target number of patients in a particular age group was not met, we overenrolled in the other group to meet the target of four patients."
Ethics oversight	The surveillance protocol was approved by the Research Review Committee and Ethical Review Committee of icddr,b.

Note that full information on the approval of the study protocol must also be provided in the manuscript.

Field-specific reporting

Please select the one below that is the best fit for your research. If you are not sure, read the appropriate sections before making your selection.

☒ Life sciences ☐ Behavioural & social sciences ☐ Ecological, evolutionary & environmental sciences

For a reference copy of the document with all sections, see nature.com/documents/nr-reporting-summary-flat.pdf

Life sciences study design

All studies must disclose on these points even when the disclosure is negative.

Sample size	No sample size calculations were conducted. Stool samples were included opportunistically according to culture result, and according to patient recruitment criteria outlined above. These samples are sufficient to provide a cross section of the V. cholerae circulating in Cox's Bazar. Four patients who met the case definition and and had no other severe comorbidity (eg, severe acute respiratory illness, acute cardiovascular symptoms, or severe acute neurological disorder) were enrolled by the physician from Saturday to Wednesday each week. Two patients with diarrhea aged less than five years old and two patients aged five years or older were enrolled each day; if the target number of patients in a particular age group was not met, we over-enrolled in the other group to meet the target of four patients. As many isolates over a set time period as possible were selected for sequencing.
Data exclusions	No data were excluded
Replication	This was a sequencing study using a unique set of samples, so replication of the sample set is not possible. However, it's important to note that the overall population structure we observed is similar to those observed in recent studies, and this is noted in the paper.
Randomization	Allocation was not random as described above in recruitment. Age distribution was not random: two patients each under and over 5 years old were attempted to be recruited, although this was not always possible.
Blinding	Binding was not possible due to the nature of recruitment (patients with acute watery diarrhoea who then tested positive for Vibrio cholerae). However, at the sequencing and analysis stages, technicians and researchers were blinded to the group allocation until the metadata was incorporated into the phylogenetic tree.

Reporting for specific materials, systems and methods

We require information from authors about some types of materials, experimental systems and methods used in many studies. Here, indicate whether each material, system or method listed is relevant to your study. If you are not sure if a list item applies to your research, read the appropriate section before selecting a response.

Materials & experimental systems

n/a	Involved in the study	
☒	☐	Antibodies
☒	☐	Eukaryotic cell lines
☒	☐	Palaeontology and archaeology
☒	☐	Animals and other organisms
☒	☐	Clinical data
☒	☐	Dual use research of concern

Methods

n/a	Involved in the study	
☒	☐	ChIP-seq
☒	☐	Flow cytometry
☒	☐	MRI-based neuroimaging
